# Adipocytes Promote Early Steps of Breast Cancer Cell Dissemination *via* Interleukin-8

**DOI:** 10.3389/fimmu.2018.01767

**Published:** 2018-07-30

**Authors:** Gabriela Vazquez Rodriguez, Annelie Abrahamsson, Lasse Dahl Ejby Jensen, Charlotta Dabrosin

**Affiliations:** ^1^Department of Oncology, Linköping University, Linköping, Sweden; ^2^Department of Clinical and Experimental Medicine, Linköping University, Linköping, Sweden; ^3^Department of Medical and Health Sciences, Division of Cardiovascular Medicine, Linköping University, Linköping, Sweden

**Keywords:** breast cancer, microdialysis, zebrafish, angiogenesis, inflammation

## Abstract

Fat is a major tissue component in human breast cancer (BC). Whether breast adipocytes (BAd) affect early stages of BC metastasis is yet unknown. BC progression is dependent on angiogenesis and inflammation, and interleukin-8 (IL-8) and vascular endothelial growth factor (VEGF) are key regulators of these events. Here, we show that BAd increased the dissemination of estrogen receptor positive BC cells (BCC) *in vivo* in the zebrafish model of metastasis, while dissemination of the more aggressive and metastatic BCC such as estrogen receptor negative was unaffected. While anti-VEGF and anti-IL-8 exhibited equal inhibition of angiogenesis at the primary tumor site, anti-IL-8 reduced BCC dissemination whereas anti-VEGF had minor effects on this early metastatic event. Mechanistically, overexpression of cell-adhesion molecules in BCC and neutrophils *via* IL-8 increased the dissemination of BCC. Importantly, the extracellular *in vivo* levels of IL-8 were 40-fold higher than those of VEGF in human BC. Our results suggest that IL-8 is a clinical relevant and promising therapeutic target for human BC.

## Introduction

Breast cancer (BC) is the most common type of cancer among women worldwide and despite increased survival rates of this disease, almost one-third of the patients will eventually develop metastatic disease (1). At this stage, treatments can only prolong life. Thus, uncovering the mechanism that facilitates BC metastasis is a clinical need. The metastatic capacity of BC cells (BCC) is governed by events in the local tumor microenvironment where the cell–cell communication takes place. This interaction is dependent on molecules released into the interstitial fluid by all different cell types in the tissue. Adipocytes are a major component of breast tissue and important contributors to regulatory interstitial proteins.

The invasive capacity of BCC is determined by events in the microenvironment such as angiogenesis and inflammation (2, 3). Two key regulators of these events are interleukin-8 (IL-8) or CXCL8 and vascular endothelial growth factor (VEGF) (4, 5, 6). We have previously reported increased levels, and an estrogen dependent regulation of both proteins in human BC and normal human breast tissue *in vivo* (7, 8, 9). Regarding the ability to induce angiogenesis, both proteins exert equal potency on endothelial cell proliferation, migration, and tube formation (10). Although VEGF overexpression has been reported to correlate with cancer progression, anti-angiogenic therapies targeting the VEGF pathways have shown little or no effect in overall survival and progression-free survival in patients with metastatic BC (11).

Interleukin-8 is a pro-inflammatory cytokine and the primary cytokine for the recruitment of neutrophils into damaged tissue (4), and we have recently reported that neutrophils play a key role in early stages of BC metastasis (12). IL-8 has also been discovered as a blood biomarker of tumor progression (13, 14). *In vitro*, IL-8 has been shown to promote cancer cell migration *per se* by up-regulating the expression of integrins (15, 16). Integrins such as vascular cell adhesion molecule 1 (VCAM-1) and intercellular adhesion molecule 1 (ICAM-1) have been shown to be involved in metastasis and cancer cell migration (17, 18). Additionally, mucin-1 (MUC-1), commonly used as a biomarker to evaluate BC recurrence and treatment response (19), has been suggested to mediate cancer cell dissemination. How these molecules are regulated by signals in the tissue microenvironment are not fully understood.

Here, we hypothesized that the release of IL-8 and VEGF by breast adipocytes (BAd) affects early metastatic event in BC.

We show that in 3D cultures *in vitro*, the secretion of IL-8 increased whereas the VEGF levels decreased by BAd. *In vivo*, in a zebrafish xenograft model, BAd significantly increased angiogenesis at the primary tumor site and enhanced the dissemination of BCC. Anti-VEGF and anti-IL-8 treatment decreased angiogenesis with equal potency in the primary tumor. However, while anti-IL-8 treatment significantly reduced BCC dissemination, anti-VEGF treatment exhibited minor effects of this early metastatic event. Low metastatic estrogen receptor positive (ER+) BCC became highly metastatic in the presence of BAd whereas both ER+ and estrogen receptor negative (ER−) BCC with intrinsically higher metastatic capacity were unaffected by BAd. Estrogen exposure further increased the dissemination of ER+ BCC.

Interleukin-8, released by BAd induced a pro-tumoral activation of neutrophils and neutrophils further increased the dissemination capacity of BCC. Furthermore, IL-8 up-regulated cell-adhesion molecules of the BCC, affecting the metastatic capacity of these cells *per se*. In human BC, the extracellular *in vivo* levels of IL-8 were 40 times higher than those of VEGF. Taken together our data suggest that BAd modify the BC microenvironment toward a pro-inflammatory and pro-angiogenic state and that IL-8 may be a clinically relevant therapeutic target.

## Materials and Methods

### Reagents

Dispase (# 17105-041), Hank’s balanced salt solution (# 14025092), DMEM (# 11880), Opti-MEM (# 11058-021), DMEM/F12 (# 11039), DMEM with high glucose (# 41965039), Opti-MEM (# 51985-042), glutamine (# 25030), penicillin-G/streptomycin (# 15070), fetal bovine serum (FBS) (# 10270), and charcoal-stripped FBS (# 12676-029) were purchased from Gibco™ (MA, USA). Bovine serum albumin (# 1.12018.0025) was purchased from Merck (NJ, USA). Apo-transferrin (# T2036), collagenase (# C7657), dexamethasone (# D4902), 3-Isobutyl-1-methylxanthine (# I7018), indomethacin (# I7378), extracellular matrix (ECM) gel (# E1270), β-estradiol (E2) (# 2758), Tricaine or MS-222 (# E10521), and insulin (# I5500) were purchased from Sigma (MO, USA). Lipofectamine RNAiMAX transfection reagent (# 13778-150), heat-inactivated FBS (# 16140-071), and ethylenediaminetetraacetic acid (EDTA; # AM9260G) were purchased from Invitrogen (MA, USA). Mammocult culture medium (# 05620) was purchased from Stem Cell Technologies Inc. (VBC, Canada). Recombinant human IL-8 (rhIL-8; # 618-IL) was purchased from R and D Systems (MN, USA). Silencer select negative control (# 4390843) and the IL-8 silencer predesigned siRNA (# AM16708) were purchased from Ambion (TX, USA). Restore™ plus western blot stripping buffer (# 46430), Fast DiI™ oil red dye (# 1635639), and DiB dye (# 60036) were purchased from ThermoFisher Scientific (MS, USA) and Biotium (CA, USA), respectively. SlowFade Gold antifade reagent with DAPI (# S36938) was purchased from Life Technologies (CA, USA). Ficoll-Paque Plus (# 17-1440-02) was purchased from GE Healthcare (IL, USA).

### Microdialysis of Patients

Women diagnosed with BC, *n* = 17, underwent microdialysis before surgery. Characteristics of the study cohorts and cancers are shown in Table 1. Cohort 1 was collected during, 2005 and 2008 and cohort 2 was collected during 2012–2015. Before insertion, 0.5 ml of lidocaine (10 mg/ml) was administrated intracutaneously. One microdialysis catheter (71/M Dialysis AB, Stockholm, Sweden) was inserted intratumorally into the BC, and another catheter was inserted into adjacent normal breast tissue and connected to a pump (CMA 107; CMA Microdialysis AB # P000127) and perfused with 154 mmol/l NaCl and 60 g/l hydroxyethyl starch (Voluven^®^; Fresenius Kabi, Uppsala, Sweden) at 0.5 µl/min. After 60-min equilibration, the perfusate was stored at −70°C.

**Table 1:** Characteristics of patients subjected to intratumoral microdialysis.

Patient	Age (years)	Tumor size	Grade (NHG)	ER (%)	PR (%)
**Cohort # 1**					
1	67	18	2	>50	>50
2	59	10	2	>50	10
3	51	20	3	>50	0
4	86	60	3	>50	>50
5	83	20	2	>50	>50
6	66	20	2	>50	0

**Cohort # 2**					
1	70	22	2	>50	>50
2	68	24	2	>50	>50
3	52	25	3	>50	10–50
4	78	28	2	>50	>50
5	62	21	2	>50	>50
6	45	40	3	0	0
7	61	25	2	>50	>50
8	48	30	2	>50	>50
9	73	30	2	>50	< 5
10	57	27	1	>50	>50
11	66	60	2	>50	>50

*One catheter was inserted within the breast cancer, and the another one was inserted in adjacent normal breast tissue the day before surgery. All cancers were HER-2 negative*.

*ER, estrogen receptor; PR, progesterone receptor*.

### Cell Lines and Primary Cultures

The ER+ cell lines MCF-7 (Cat # HTB-22, RRID:CVCL_0031) and T47D (Cat # HTB-133, RRID:CVCL_0553) and the ER− cell line MDA-MB-231 (Cat # HTB-26, RRID:CVCL_0062) were purchased from American Type Culture Collection (ATCC) (Manassas, VA, USA) and authenticated by ATCC. MCF-7 and MDA-MB-231 were maintained in DMEM supplemented with 2 mM glutamine, penicillin-G/streptomycin 50 IU/ml/50 μg/ml and 10% FBS. T47D cells were cultured in Opti-MEM with penicillin-G/streptomycin 50 IU/ml/50 μg/ml and 4% FBS. For treatments, ER+ BCC were cultured in DMEM/F12 medium with 0.02% of BSA, 10 µg/ml apo-transferrin, and 1 µg/ml insulin.

#### Culture of Human BAd

Fat tissue from normal human breast tissue was collected in PBS containing pENICILLIN-G/streptomycin 25 IU/ml/25 μg/ml and 10% FBS. Fat tissue was washed with PBS and cleared from blood vessels and cut in small pieces. Minced tissue was placed in a 50 ml falcon tube with dissociation solution (0.25 mg/ml collagenase and 2.5 mg/ml dispase in Hank’s balanced salt solution) and incubated at 37°C for 30 min. Pipetting several times during incubation helped to further dissociate the cells. Cells were washed with PBS/2% FBS and centrifuged twice at 200 × *g* for 5 min. Breast pre-adipocytes were cultured in high glucose DMEM supplemented with 2 mM glutamine, penicillin-G/streptomycin 50 IU/ml/50 μg/ml, and 10% FBS.

For adipogenic differentiation, cells were cultured 5 or 12 days where specified in DMEM with 10% FBS, penicillin-G/streptomycin 50 IU/ml/50 μg/ml, dexamethasone 1 µM, 3-isobutyl-1-methylxanthine 0.5 mM, insulin 50 µg/ml, and indomethacin 200 µM. Cells were stained with red oil, Oil red O (# O0625), 30 mi on 4% PFA-fixed adipocytes. Pictures were taken with an Olympus BX43 light/fluorescence microscope (20×/0.50 magnification), using an Olympus DP72 CCD camera. Images were acquired with the Olympus CellSens Imaging software version 1.16 (Olympus cellSens Software, RRID:SCR_016238).

Collected conditioned medium from BAd was obtained as follows: breast pre-adipocytes were differentiated, washed, and then cultured in DMEM with 10% charcoal stripped FBS, 2 mM glutamine, and penicillin-G/streptomycin 50 IU/ml/50 μg/ml 24 h at 37°C and 5% CO_2_. Only medium without cells served as control.

#### Culture of Human Neutrophils

After informed consent, neutrophils were isolated from venous blood collected from female donors. In brief, blood sample was diluted 1:3 in PBS/2 mM EDTA with 0.1% heat-inactivated FBS and separated by Ficoll-Paque gradient and red blood cell pellets containing neutrophils were diluted and sedimentated 20 min in 3% dextran T-500/0.9% NaCl. Residual red blood cells in the collected neutrophils were lysed in hypotonic saline solutions. For *in vitro* and *in vivo* experiments, neutrophils were re-suspended in DMEM/F12 with 0.02% of BSA, 10 µg/ml apo-transferrin and 1 µg/ml insulin.

For immunocytochemistry, neutrophils were diluted in BAd-conditioned or control medium, incubated at 37°C during 45 min with rabbit anti-human IL-8 (Bio-Rad/AbD Serotec Cat # AHP781, RRID:AB_2264854) or rabbit isotype control (Novus Biologicals # NBP2-24893) on 19 mm cover glasses in 12-well plates. Neutrophils were counted before and after treatments with Trypan Blue viability exclusion dye.

### Co-Cultures and 3D Cultures of BAd and BCC

For monolayer co-cultures, breast pre-adipocytes were differentiated for 5 days before MDA-MB-231, MCF-7, and T47D cells were added at 3 × 10^3^ or 4 × 10^3^ cells/well. After 24 h co-culture, treatment ± E2 1 nM, rabbit anti-human IL-8, rabbit anti-human VEGF (Acris Antibodies GmbH Cat # PP1073P1, RRID:AB_1008432), or rabbit isotype antibodies at 1 µg/ml was performed for 3 days.

For 3D cultures, mammospheres with MDA-MB-231, MCF-7, and T47D cells were cultured either alone or in combination with breast pre-adipocytes in 96-well plates. MCF-7 and T47D were cultured in Mammocult medium, and MDA-MB-231 was cultured in high-glucose DMEM supplemented with 2 mM glutamine, penicillin-G/streptomycin 50 IU/ml/50 μg/ml, 10% FBS, and 2.5% ECM gel. Mammospheres with pre-adipocytes were differentiated for 5 days and then treated ± E2 1 nM for 7 days.

### RNA Interference Experiments

MDA-MB-231 cells were seeded at 2 × 10^5^ cells/well in six-well plates in DMEM supplemented with 10% FBS and 2 mM glutamine. After 24 h incubation, fresh medium was added with 25 pmol of the silencer select negative control or the IL-8 silencer predesigned siRNA and 7.5 µl of lipofectamine RNAiMAX transfection reagent. All reagents were prepared in Opti-MEM medium. After 48 h incubation, culture media were collected for the ELISA measurement of secreted IL-8 to evaluate the efficiency of the transfection. Cells were either lysed in RIPA buffer for western blot analysis or labeled with Fast DiI™ oil red dye, as previously described (20), for zebrafish experiments.

### Immunocytochemistry

Neutrophils and BAd cultured on cover glasses were fixed in 4% PFA and exposed to anti-human lymphocyte function-associated antigen 1 (LFA-1) 1:100 (BioLegend Cat # 301213, RRID:AB_314151), anti-human perilipin A 1:200 (Abcam Cat # ab3526, RRID:AB_2167274), Alexa Fluor – 488 1:500 (Abcam Cat# ab150113, RRID:AB_2576208), and Alexa Fluor – 546 1:500 (Thermo Fisher Scientific Cat # A-11010, RRID:AB_2534077), mounted in SlowFade Gold antifade reagent with DAPI and visualized using an Olympus BX43 light/fluorescence microscope (40×/0.75 magnification) with excitation filters BP360-370, BP530-550, and BP460-495, using an Olympus DP72 CCD camera. Images were acquired with the Olympus CellSens Imaging software version 1.16 (Olympus cellSens Software, RRID:SCR_016238). A number of positive stained cells were quantified using the Fiji software (Fiji, RRID:SCR_002285).

### Cytokine Quantification

Interleukin-8, VEGF, CCL2, and CCL5 were quantified by Human Fluorokine MAP base kits with corresponding beads (R and D Systems #LUH000) and analyzed with Luminex^®^ 200™ (Luminex, Austin, TX, USA). Secreted leptin in culture medium from differentiated BAd was quantified by ELISA (R and D Systems #DLP00). Secreted IL-8 from transfected MDA-MB-231 cells, BAd-conditioned, and control medium was quantified by ELISA (R and D Systems #Q8000B). Microdialyzates were also analyzed using the proximity extension assay (PEA) technology (Olink AB, Uppsala, Sweden).

### Migration Assay

MCF-7, T47D, and MDA-MB-231 cells were seeded at 1 × 10^4^ cells/well alone or in combination with breast pre-adipocytes in a CytoSelect 96-well cell migration assay plate (Cell Biolabs #CBA-106) in DMEM/F12 with 0.02% BSA, 10 µg/ml apotransferrin and 1 µg/ml insulin. Complete DMEM supplemented with 2 mM glutamine, penicillin-G/streptomycin 50 IU/ml/50 μg/ml, and 10% FBS was used as chemoattractant in the lower chamber. Provider’s instructions were followed, and cell migration was analyzed using the Spark™ 10 M (Tecan Trading AG, Switzerland).

### Western Blot

Cell lysates were loaded into 4–15% SDS-PAGE gels (BioRad # 4561083) and transferred to PVDF membranes (BioRad # 170-4156). Primary antibodies, mouse anti-human VCAM-1 1:1,000 (Novus Cat # NBP1-28404, RRID:AB_1852860), mouse anti-human ICAM-1 1 µg/ml (Novus Biologicals # NBP2-22541), mouse anti-human MUC-1 1 µg/ml (Novus Biologicals # NBP2-34737), and rabbit anti-human GAPDH 1:2,500 (Abcam Cat # ab9485, RRID:AB_307275) were followed by goat anti-rabbit HRP 1:2,000 (Dako Cat # P0448, RRID:AB_2617138) and goat anti-mouse HRP 1:2,500 (Dako Cat # P0447, RRID:AB_2617137). Membranes were stripped by using the restore plus western blot stripping buffer (Thermo Scientific) for the analysis of GAPDH expression by following the manufacturer’s instructions. Membranes were revealed with ECL prime western blotting detection reagent (GE Healthcare # RPN2232), visualized by using the Chemidoc™ MP Imaging System and analyzed with Image Lab™ Software version 5.2.1 (Image Lab Software, RRID:SCR_014210). Western blots shown in figures were prepared by cropping and pasting from original membranes shown in full in Figure S1 in Supplementary Material.

### Zebrafish Tumor Xenograft Model

MCF-7 and T47D cells were cultured ± E2 1 nM for 48 h, all BCC were labeled with 4 µg/ml Fast DiI™ oil red dye as previously described (20), and neutrophils were labeled with 6 µg/ml DiB. BCC were injected ±50% of BAd or ±33% BAd + 33% neutrophils. rhIL-8 was added at 1 µg/ml, and rabbit anti-human IL-8, rabbit anti-human VEGF, goat anti-human CCL2 (R and D Systems Cat# AB-279-NA, RRID:AB_354333), goat anti-human CCL5 (R and D Systems Cat# AB-278-NA, RRID:AB_354332), mouse anti-human LFA-1 (Biolegend), rabbit isotype control, goat isotype control (R and D Systems Cat# AB-108-C, RRID:AB_354267), or mouse isotype control (BioLegend Cat# 401408, RRID:AB_11148942) antibodies were added at 0.1 mg/ml ± E2 1 nM immediately before injections.

Transgenic Tg(fli1:EGFP)^y1^ zebrafish embryos, with fluorescent green blood vessels, were raised in E3 medium supplemented with 0.2 mM 1-phenyl-2-thiourea (PTU) at 28°C, and 2 days old zebrafish embryos were used for cell implantation into the perivitelline space, as previously described (20). After cell injections, zebrafish embryos were selected under fluorescence, only zebrafish embryos with correctly injected cells were included in the experiments, embryos with cells present in the yolk sac were discarded. Embryos were incubated at 28°C in E3 medium with 0.2 mM PTU and ±E2 1 nM where indicated. Cells dissemination to the zebrafish’s tail region was evaluated at 1 or 3 days post-injection. Co-disseminated BCC/neutrophils in the tail’s zebrafish region were quantified at 1 day post-injection. The embryos were anesthetized with 0.02% MS-222 and pictures were taken in an Olympus BX43 light/fluorescence microscope (10×/0.30 magnification) with excitation filters BP360-370, BP460-495, and BP530-550, using an Olympus DP72 CCD camera. Images were acquired with the Olympus CellSens Imaging software version 1.16. To evaluate tumor angiogenesis, zebrafish embryos were fixed in paraformaldehyde 4% overnight at 4°C, washed with PBS and mounted in SlowFade Gold antifade reagent with DAPI. Confocal images of intratumor vasculature were obtained in an upright confocal microscope Zeiss Axio imager with LSM 700 (Zeiss, Oberkochen, Germany), and the intratumor vessel area was quantified by using the Fiji software (Fiji, RRID:SCR_002285).

### Equipment and Settings

Confocal acquisition of intratumoral vasculature images of zebrafish was performed as follows: image size (pixels) 1,024 × 1,024, 16-bit depth, averaging of 4, Plan-Apochromat 20x/0.8 M27 objective was used, laser wavelength 488 and 555 nm, binning mode 1 × 1, and PMT detector. EGFP excitation and emission wavelengths were 488 and 509, respectively; and Dil excitation and emission wavelengths were 549 and 565, respectively. Laser power was used at 2%, and z-stack intervals were set up to 8 µm.

### Statistical Analysis

Data are presented as mean ± SEM. Student’s *t*-tests, Wilcoxon signed-rank test, and Spearman’s correlation test were used where appropriate. A *p* value of <0.05 was considered statistically significant. Statistics were performed with Prism 7.0 (Graphpad Prism, RRID:SCR_002798).

### Availability of Materials

All materials such as protocols, analytic methods, and study materials used to conduct this research are available to other researchers upon request.

## Results

### The Extracellular *In Vivo* Levels of IL-8 Were 40 Times Higher Than Those of VEGF in Human BC

**Figure 1:**
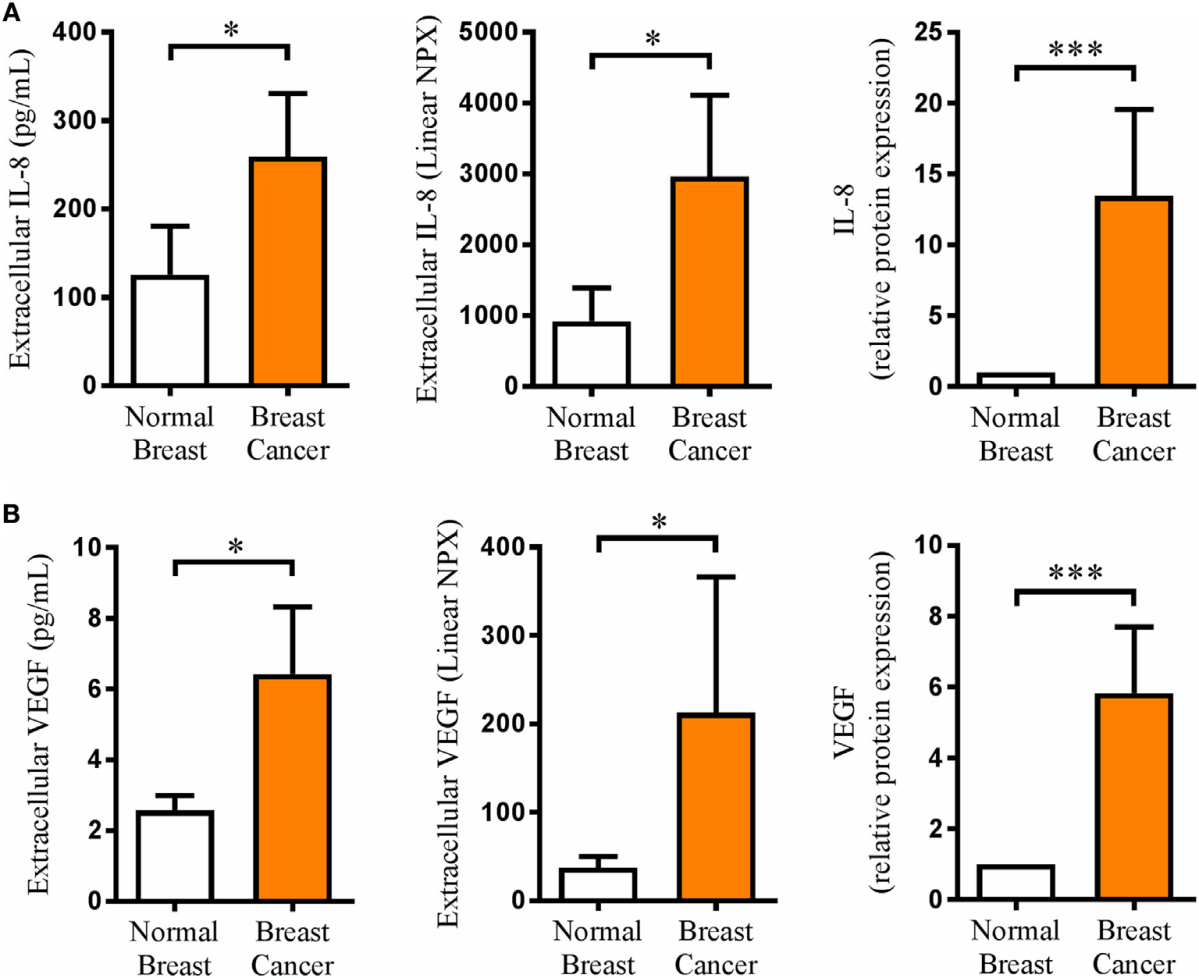
Extracellular *in vivo* levels of interleukin-8 (IL-8) and vascular endothelial growth factor (VEGF) were significantly increased in human breast cancer. A total of 17 breast cancer patients underwent microdialysis before surgery. One catheter was inserted into the breast cancer, and another catheter was inserted into adjacent normal breast tissue as control. Proteins were analyzed by Luminex in cohort 1 (expressed as pg/ml), *n* = 6 and by proximity extension assay (PEA) technology in cohort 2 (expressed as linear NPX), *n* = 11 as described in Section “Materials and Methods.” The bars to the right represent data from both cohorts normalized to the control in each cohort (relative protein expression), *n* = 17. **(A)** IL-8 *in vivo* measurements in microdialysis samples from breast cancer patients. **(B)** VEGF *in vivo* measurements in microdialysis samples from breast cancer patients. Results are presented as mean ± SEM and were analyzed pairwise by Wilcoxon signed-rank test, *p < 0.05, ***p < 0.001. IL-8 and VEGF exhibited a significant positive correlation by Spearman’s correlation test, *r* = 0.51 and *p* < 0.05.

Microdialysis, which we have pioneered for sampling of extracellular compounds in normal human breast tissue and BC (21, 22), was performed in BC patients before surgery for sampling of proteins from the interstitial fluid *in vivo* in live tissue. Two different methods were used for protein quantification. As shown in Figure 1, IL-8 and VEGF levels were significantly increased in BC compared to normal breast tissue. Furthermore, the levels of IL-8 were approximately 40 times higher than those of VEGF. Additionally, IL-8 and VEGF exhibited a significant positive correlation, *r* = 0.51 and *p* < 0.05.

### BCC/BAd Mammospheres Increased Secretion of IL-8 But Decreased VEGF Secretion

**Figure 2:**
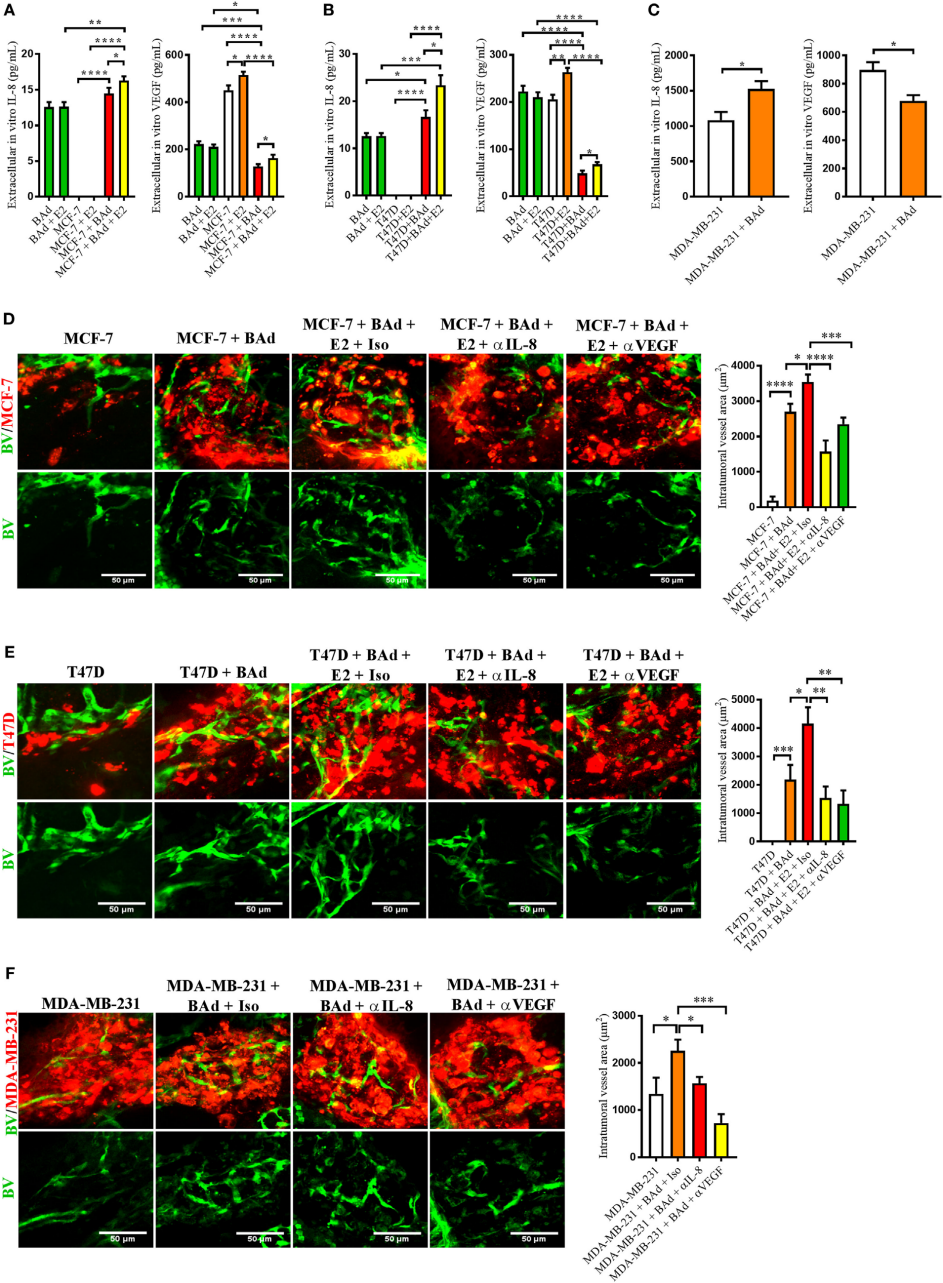
Addition of breast adipocytes (BAd) significantly increased interleukin-8 (IL-8) secretion but decreased vascular endothelial growth factor (VEGF) secretion compared to breast cancer cells (BCC) cultured alone and anti-IL-8 and anti-VEGF significantly decreased BAd-induced angiogenesis in primary tumors in zebrafish. BCC were cultured in 3D spheres alone or in combination with BAd. Secreted IL-8 and VEGF were analyzed as described in Section “Materials and Methods.” Prior injections, breast pre-adipocytes were differentiated for 12 days and estrogen receptor positive (ER+) BCC were cultured ± β-estradiol (E2) 1 nM for 48 h. All BCC were labeled with 4 µg/ml Fast DiI™ oil red dye. Cells were injected ± anti-IL-8, anti-VEGF, or isotype control at 0.1 mg/ml ± E2 1 nM into the perivitelline space of 2 days old zebrafish embryos, which expressed enhanced green fluorescent protein in endothelial cells. **(A)** BAd mammospheres alone and low metastatic ER+ MCF-7 ± 90% BAd mammospheres were cultured ± E2 1 nM during 7 days, *n* = 4–5 in each group. **(B)** BAd mammospheres alone an ER+ T47D with intrinsically higher metastatic capacity ± 90% BAd mammospheres were cultured ± E2 1 nM during 7 days, *n* = 5–6 in each group. **(C)** Estrogen receptor negative (ER−) metastatic MDA-MB-23 ± 90% BAd and BAd mammospheres were cultured during 7 days, *n* = 4–5 in each group. **(D)** MCF-7 cells were injected alone or in combination with 50% BAd ± E2 1 nM, tumor angiogenesis was analyzed 3 days post-injections, *n* = 12–18 in each group. **(E)** T47D cells were injected alone or in combination with 50% Bad ± E2 1 nM, tumor angiogenesis was analyzed 3 days post-injections, *n* = 10 in each group. **(F)** MDA-MB-231 cells were injected alone or in combination with 50% BAd, tumor angiogenesis was analyzed 3 days post-injections, *n* = 7–10 in each group. Representative confocal images are shown for each cell line. BV = blood vessels. Results are presented as mean ± SEM and analyzed by Student’s *t*-test, *p < 0.05, **p < 0.01, ***p < 0.001, ****p < 0.0001. Data are representative of at least two independent experiments.

To further investigate the role of BAd in the secretion of IL-8 and VEGF, mammospheres of different ER+ and ER− BCC were set up with or without the addition of BAd. In mammospheres with the low metastatic ER+ cells MCF-7, and in the ER+ cells with intrinsically increased metastatic capacity, T47D, and in the ER− cells with high metastatic capacity MDA-MB-231, the addition of BAd significantly increased the secreted levels of IL-8 (Figures 2A–C). Exposure to E2 further increased IL-8 and VEGF levels in the ER+ mammospheres (Figures 2A,B). Surprisingly, the addition of BAd decreased the secretion of VEGF significantly in all the BCC mammospheres tested (Figures 2A–C). The differentiation of pre-adipocytes into adipocytes was confirmed using red oil, perilipin A, and leptin secretion (Figure S2A in Supplementary Material).

### Anti-IL-8 and Anti-VEGF Treatment Reduced BAd-Induced Angiogenesis in Primary BC Tumor Xenografts with Equal Potency

To assess the role of BAd in tumor angiogenesis at the primary tumor site, ER+ MCF-7 and T47D cells or ER− MDA-MB-231 cells were injected alone or in combination with BAd in zebrafish. As shown in Figures 2D–F, tumor xenografts exhibited a significant increase in angiogenesis in the presence of BAd and this effect was further increased in the presence of E2 in ER+ BC primary tumors (Figures 2D,E). Treatment with anti-IL-8 and anti-VEGF counteracted the BAd-induced angiogenesis in all tumors (Figures 2D–F).

### Anti-IL-8 Treatment Significantly Decreased BCC Dissemination

**Figure 3:**
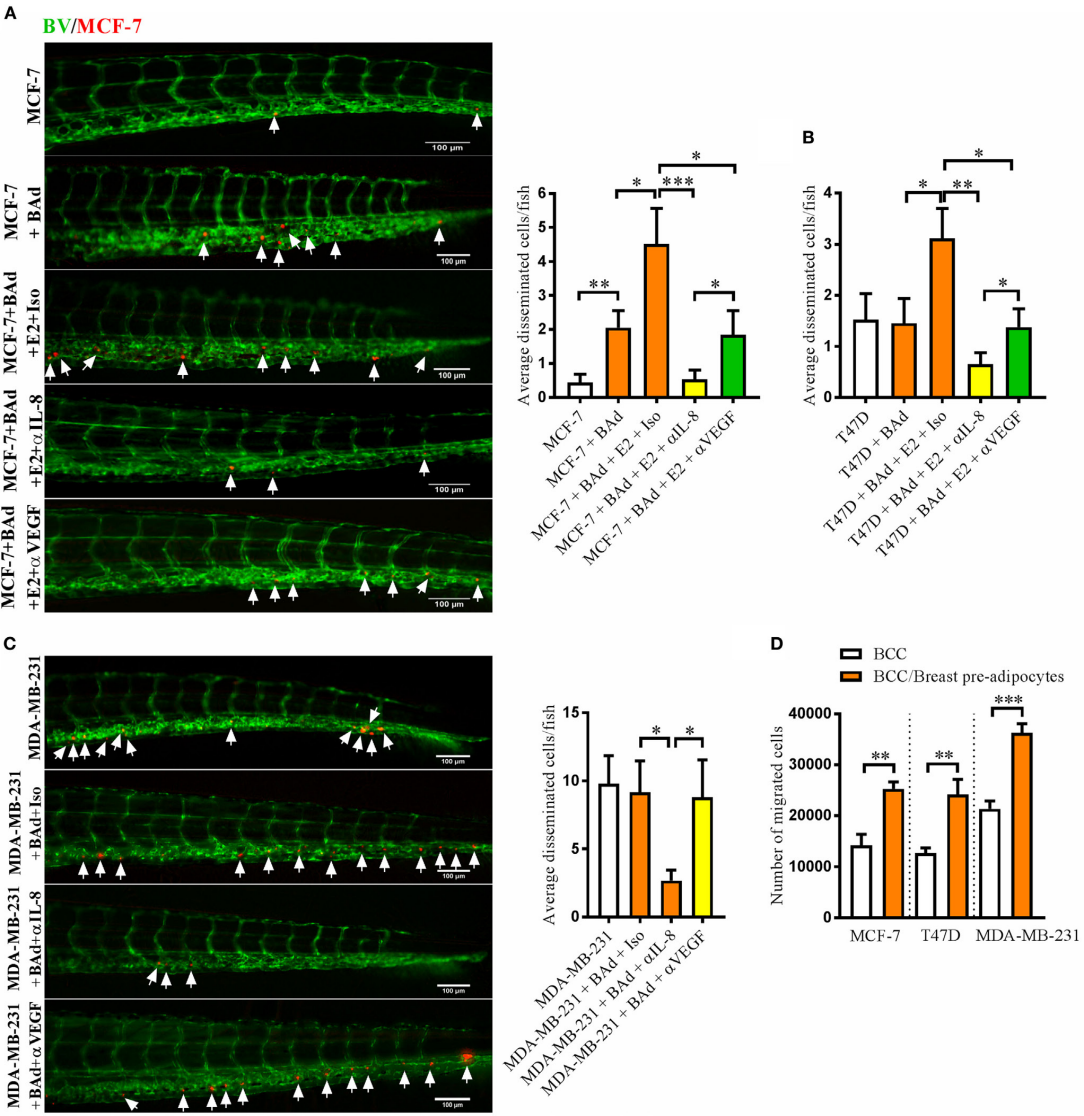
Anti-interleukin-8 (IL-8) treatment significantly decreased breast cancer cells (BCC) dissemination induced by breast adipocytes (BAd). Prior injections, breast pre-adipocytes were differentiated for 12 days and estrogen receptor positive (ER+) BCC were cultured ± β-estradiol (E2) 1 nM for 48 h. All BCC were labeled with 4 µg/ml Fast DiI™ oil red dye. Cells were injected ± anti-IL-8, anti-VEGF, or isotype control at 0.1 mg/ml ± E2 1 nM into the perivitelline space of 2 days old zebrafish embryos, which expressed enhanced green fluorescent protein in endothelial cells. **(A)** MCF-7 cells were injected alone or in combination with 50% BAd ± E2 1 nM. BCC dissemination was evaluated 3 days post-injections, *n* = 18–27 in each group. **(B)** T47D cells were injected alone or in combination with 50% BAd ± E2 1 nM. BCC dissemination was evaluated 3 days post-injections, *n* = 16–28 in each group. **(C)** MDA-MB-231 cells were injected alone or in combination with 50% BAd. BCC dissemination was evaluated 3 days post-injections, *n* = 14–18 in each group. **(D)** MCF-7, T47D, and MDA-MB-231 cells were cultured alone or in combination with 50% breast pre-adipocytes *in vitro* during 24 h, and migration of cells was determined as described in Section “Materials and Methods,” *n* = 6 in each group. BV = blood vessels. Arrows indicate disseminated BCC. Results are presented as mean ± SEM and analyzed by Student’s *t*-test, *p < 0.05, **p < 0.01, ***p < 0.001. Data are representative of at least two independent experiments.

Next, we investigated whether BAd affected the dissemination capacity of BCC and whether this effect was mediated by IL-8 or VEGF. In the presence of BAd the otherwise low metastatic ER+ MCF-7 cells exhibited increased dissemination compared to the cells injected alone and this effect was further increased in the presence of E2 (Figure 3A). The dissemination of the ER+ T47D, which are intrinsically more aggressive than the ER+ MCF-7 cells, and the ER− MDA-MB-231 cells was not affected by BAd (Figures 3B,C). However, E2 treatment significantly increased T47D cell dissemination in the presence of BAd (Figure 3B). Anti-IL-8 treatment significantly reduced the dissemination of all BCC while anti-VEGF treatment had no effect in MDA-MB-231 cell dissemination and showed less efficiency in inhibition of MCF-7 and T47D cell dissemination (Figures 3A–C). Interestingly, breast pre-adipocytes significantly increased the migration of all BCC *in vitro* (Figure 3D).

### Anti-IL-8 and Anti-VEGF Altered Cytokine Secretion in BCC/BAd Cultures, Which in Turn Affected BCC Dissemination

**Figure 4:**
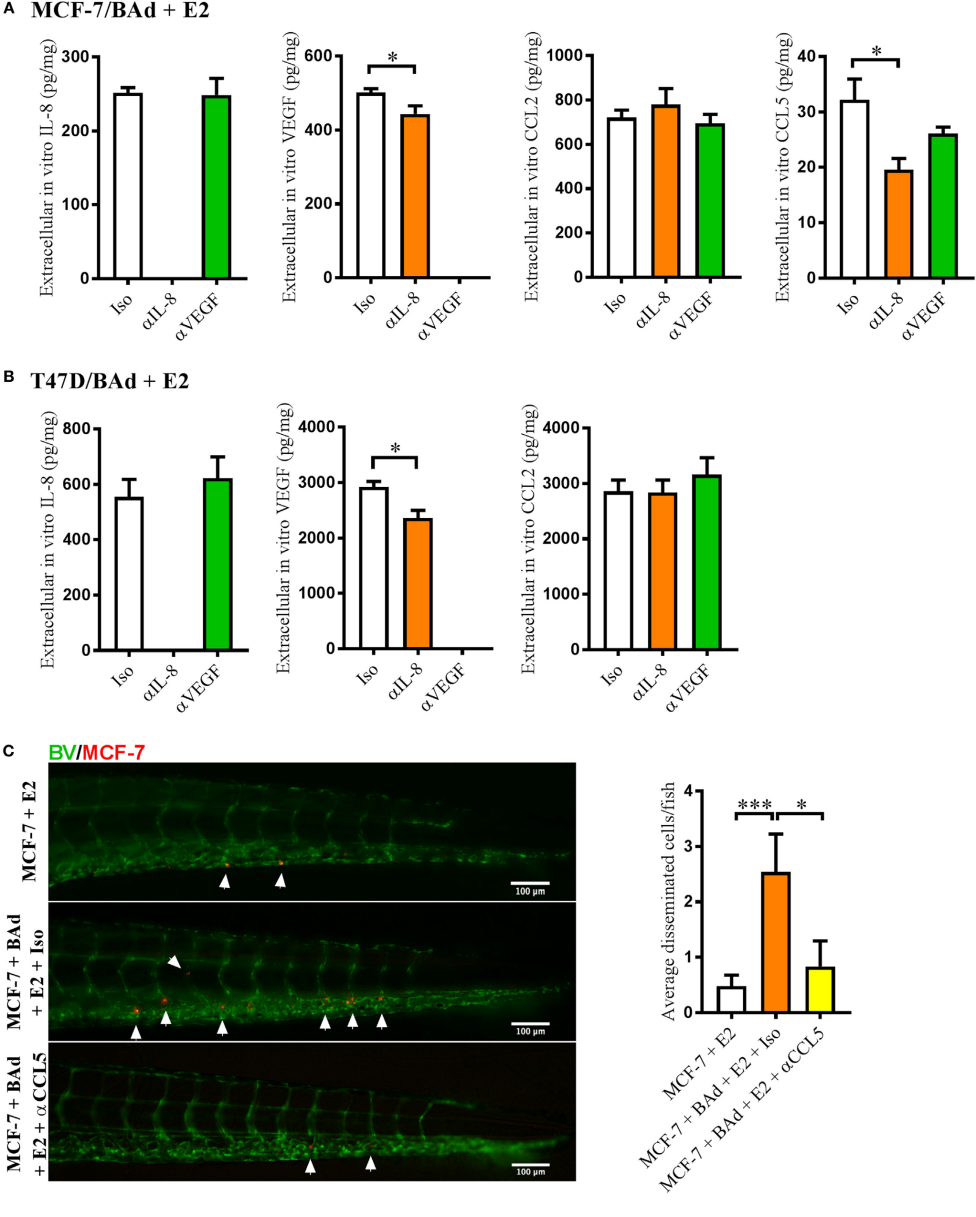
Anti-interleukin-8 (αIL-8) decreased vascular endothelial growth factor (VEGF) and CCL5 secretion, which affected estrogen receptor positive (ER+) breast cancer cells (BCC) dissemination. For monolayer co-cultures, breast pre-adipocytes were differentiated for 5 days before ER+ BCC were added at 4 × 10^3^ cells/well. For zebrafish experiments, breast pre-adipocytes were differentiated for 12 days, MCF-7 cells were cultured + β-estradiol (E2) 1 nM for 48 h and labeled with 4 µg/ml Fast DiI™ oil red dye before injected into the perivitelline space of 2 days old zebrafish embryos, which expressed enhanced green fluorescent protein in endothelial cells. **(A)** MCF-7 cells were co-cultured with 50% breast adipocytes (BAd) in the presence or absence of αIL-8, anti-VEGF (αVEGF), or control isotype (Iso) antibodies at 1 µg/ml during 3 days in the presence of E2 1 nM, and secreted cytokines were quantified as described in Section “Materials and Methods,” *n* = 5–4 in each group. **(B)** T47D cells were co-cultured with 50% BAd in the presence or absence of αIL-8, αVEGF, or control Iso antibodies at 1 µg/ml during 3 days in the presence of E2 1 nM, and secreted cytokines were quantified as described in Section “Materials and Methods,” *n* = 6–5 in each group. **(C)** MCF-7 cells were injected in zebrafish embryos alone or in combination with 50% BAd ± anti-CCL5 (αCCL5) or Iso control antibody at 0.1 mg/ml and E2 1 nM, as described in Section “Materials and Methods.” MCF-7 dissemination was analyzed 3 days post-injections, *n* = 13–27 in each group. Representative images of zebrafish embryos are shown. Arrows show disseminated BCC cells. BV = blood vessels. Results are presented as mean ± SEM, Student’s *t*-test, *p < 0.05, ***p < 0.001. Data are representative of at least two independent experiments.

To evaluate whether anti-IL-8 or anti-VEGF treatment affected the secretion of other cytokines that may be involved in BCC dissemination, we performed *in vitro* monolayer co-cultures of E2 stimulated MCF-7 and T47D + BAd or MDA-MB-231 + BAd in the presence of anti-IL-8 or anti-VEGF antibodies. Anti-IL-8 treatment significantly decreased the secretion of CCL5 and VEGF in MCF-7/BAd co-cultures (Figure 4A). The role of CCL5 in the dissemination process was confirmed in zebrafish experiments, which revealed that MCF-7 cell dissemination in the presence of BAd could be counteracted by anti-CCL5 treatment (Figure 4C). In T47D/BAd co-cultures, the anti-IL8 treatment reduced the secretion of VEGF, while CCL5 was undetectable (Figure 4B). Anti-VEGF treatment showed no effect in secreted cytokines levels in any of the ER+ BCC/BAd co-cultures (Figures 4A,B).

**Figure 5:**
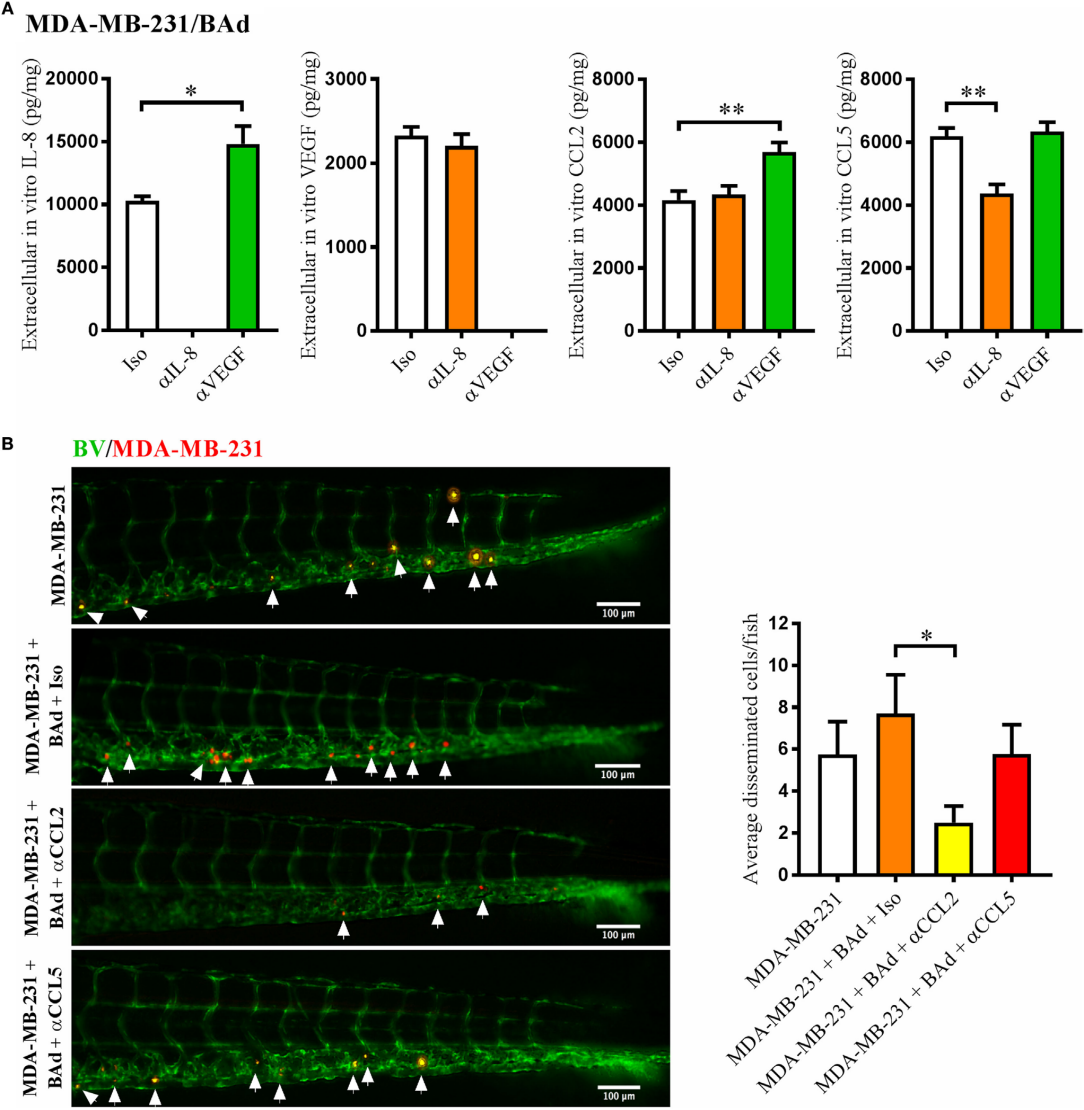
Anti-interleukin-8 (αIL-8) decreased CCL5 and anti-vascular endothelial growth factor (αVEGF) increased interleukin-8 (IL-8) and CCL2, which affected the dissemination of estrogen receptor negative (ER−) breast cancer cells (BCC). For monolayer co-cultures, breast pre-adipocytes were differentiated for 5 days before MDA-MB-231 cells were added at 3 × 10^3^ cells/well. For zebrafish experiments, breast pre-adipocytes were differentiated for 12 days and MDA-MB-231 cells were labeled with 4 µg/ml Fast DiI™ oil red dye before injected into the perivitelline space of 2 days old zebrafish embryos, which expressed enhanced green fluorescent protein in endothelial cells. **(A)** MDA-MB-231 cells were co-cultured with 50% breast adipocytes (BAd) in the presence or absence of αIL-8, αVEGF, or control isotype (Iso) antibodies at 1 µg/ml during 3 days, and secreted cytokines were quantified as described in Section “Materials and Methods,” *n* = 4 in each group. **(B)** MDA-MB-231 cells were injected in zebrafish embryos alone or in combination with 50% BAd ± anti-CCL2 (αCCL2), anti-CCL5 (αCCL5), or control Iso antibodies at 0.1 mg/ml, as described in Section “Materials and Methods.” BCC dissemination was analyzed 3 days post-injections, *n* = 20–24 in each group. Representative images of zebrafish embryos are shown. Arrows show disseminated BCC. BV = blood vessels. Results are presented as mean ± SEM, Student’s *t*-test, *p < 0.05, **p < 0.01. Data are representative of at least two independent experiments.

Similar to the MCF-7 cells, anti-IL-8 treatment decreased the secretion of CCL5 in MDA-MB-231/BAd co-cultures, while anti-VEGF treatment increased the secretion of IL-8 and CCL2 (Figure 5A). Zebrafish experiments showed that MDA-MB-231 cell dissemination in the presence of BAd was significantly reduced with an anti-CCL2, but not with anti-CCL5 treatment (Figure 5B).

### Increased LFA-1 Expression in Neutrophils by Conditioned Medium from BAd Was Significantly Reduced in Presence of Anti-IL-8

**Figure 6:**
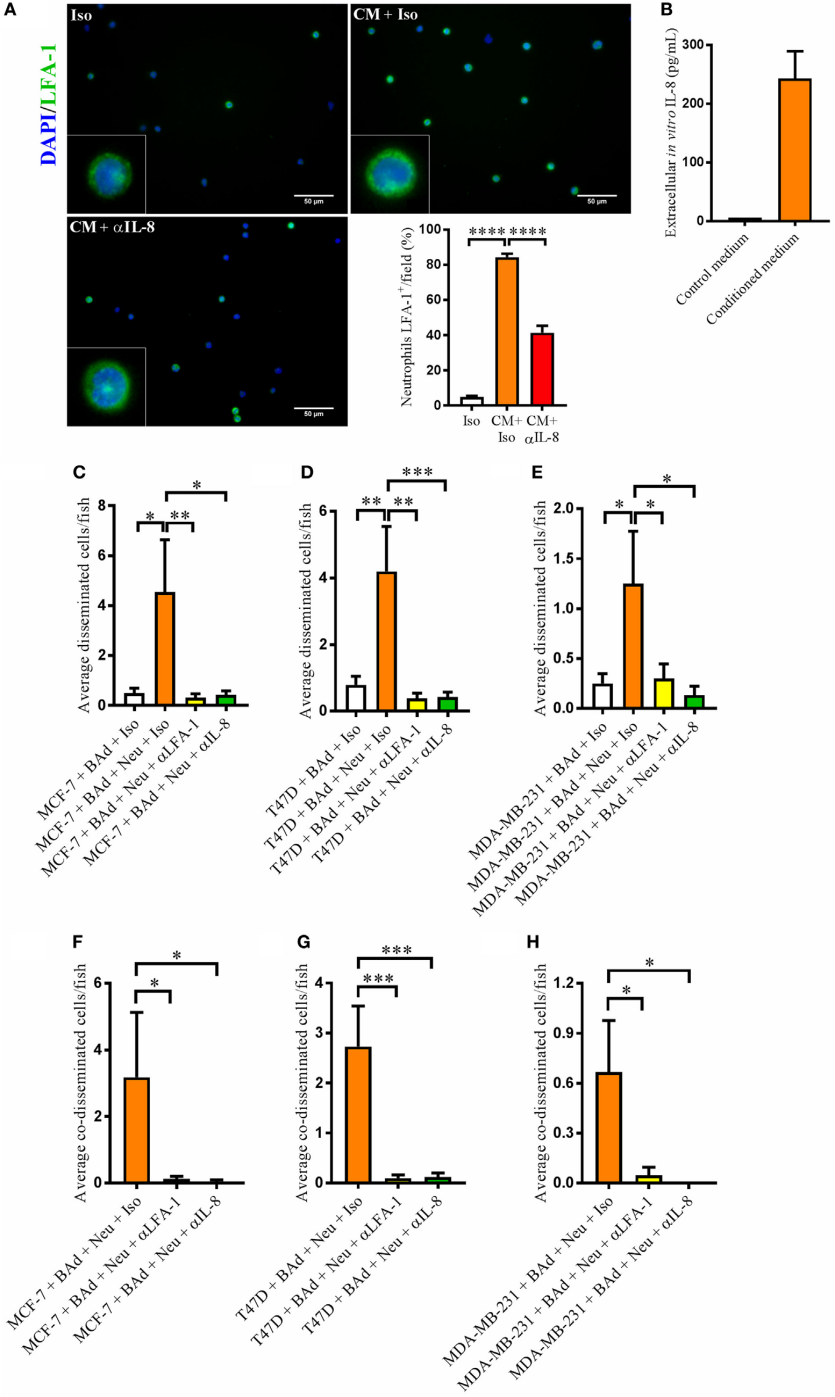
Anti-interleukin-8 (αIL-8) reduced lymphocyte function-associated antigen 1 (LFA-1) expression in neutrophils and the neutrophil-mediated dissemination of breast cancer cells (BCC) in the presence of breast adipocytes (BAd). For immunocytochemistry, neutrophils were cultured at 1 × 10^6^ cells/ml in BAd-conditioned or control medium and incubated 45 min at 37°C. Prior zebrafish injections, breast pre-adipocytes were differentiated for 12 days, BCC were labeled with 4 µg/ml Fast DiI™ oil red dye, and neutrophils were labeled with 6 µg/ml DiB. All BCC were injected ± αIL-8, anti-LFA-1 (αLFA-1), or isotype (Iso) control antibodies at 0.1 mg/ml into the perivitelline space of 2 days old zebrafish embryos, which expressed enhanced green fluorescent protein in endothelial cells. **(A)** Neutrophils were cultured ± conditioned medium (CM) from BAd ± αIL-8 or Iso control at 1 µg/ml, and whole cells were stained with anti-human LFA-1 as described in Section “Materials and Methods” (*n* = 15 random fields per group). Insets show magnification of the cells. **(B)** Breast pre-adipocytes were differentiated during 12 days and cultured in DMEM supplemented medium during 24 h, and secreted interleukin-8 (IL-8) was measured in control and CM as described in Section “Materials and Methods,” *n* = 4 in the CM group. **(C)** MCF-7 cells were injected in zebrafish embryos together with 33% BAd ± 33% neutrophils, as described in Section “Materials and Methods.” BCC dissemination was analyzed at 1 day post-injections, *n* = 11–25 in each group. **(D)** T47D cells were injected in zebrafish embryos together with 33% BAd ± 33% neutrophils, as described in Section “Materials and Methods.” BCC dissemination was analyzed at 1 day post-injections, *n* = 15–26 in each group. **(E)** MDA-MB-231 cells were injected in zebrafish embryos together with 33% BAd ± 33% neutrophils, as described in Section “Materials and Methods.” BCC dissemination was analyzed at 1 day post-injections, *n* = 12–21 in each group. **(F)** Number of co-disseminated MCF-7/neutrophil cells was quantified as described in Section “Materials and Methods,” *n* = 17–21 in each group. **(G)** Number of co-disseminated T47D/neutrophil cells was quantified as described in Section “Materials and Methods,” *n* = 21–26 in each group. **(H)** Number of co-disseminated MDA-MB-231/neutrophil cells was quantified as described in Section “Materials and Methods,” *n* = 12–21 in each group. Results are presented as mean ± SEM, Student’s *t*-test, *p < 0.05, **p < 0.01, ***p < 0.001, ****p < 0.0001. Data are representative of at least two independent experiments.

Interleukin-8 is a key neutrophil chemotactic factor. As neutrophils may alter their phenotype into a pro- or anti-tumoral state, we analyzed whether conditioned medium from BAd was able to stimulate such phenotypic alteration. LFA-1 integrin is involved in the first steps of cell adhesion and neutrophil recruitment to the tumor (23). As shown in Figure 6A, neutrophils treated with conditioned medium from BAd, which contained high levels of IL-8 (Figure 6B), increased the LFA-1 expression significantly, whereas anti-IL-8 treatment reduced this expression. Viability of neutrophils was not altered in any experimental group after treatments (Figure S2B in Supplementary Material).

### Neutrophils Increased BCC Dissemination in Presence of BAd, Which Was Significantly Reduced by Anti-IL-8 and Anti-LFA-1

Lymphocyte function-associated antigen 1 integrin is an important mediator of cell–cell interactions involved in neutrophil-mediated BCC intra- and extravasation. Therefore, we investigated whether neutrophils affected BCC dissemination in the presence of BAd. Addition of neutrophils further increased the dissemination of ER+ and ER− BCC/BAd in the zebrafish and this effect decreased significantly in the presence of anti-LFA-1 and anti-IL-8 treatments (Figures 6C–E). Anti-LFA-1 and anti-IL-8 also reduced the BCC/neutrophils co-dissemination (Figures 6F–H).

### IL-8 Mediated BCC Dissemination and Modified the Expression of Cell-Adhesion Molecules

**Figure 7:**
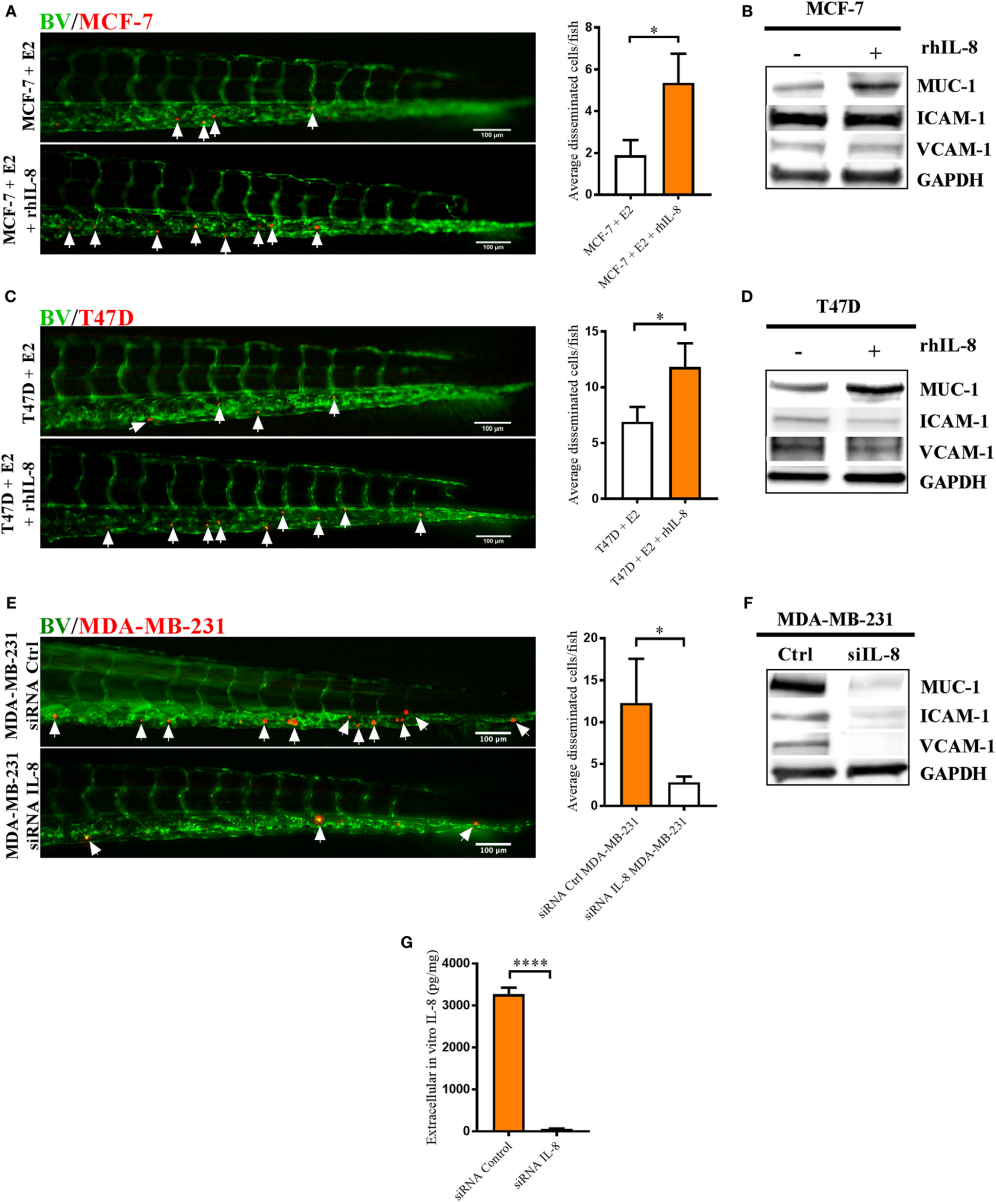
Interleukin-8 (IL-8) increased dissemination and MUC-1 expression in estrogen receptor positive (ER+) breast cancer cells (BCC) and IL-8 gene silencing affected the dissemination and expression of intercellular adhesion molecule 1 (ICAM-1), vascular cell adhesion molecule 1 (VCAM-1), and mucin-1 (MUC-1) integrins in estrogen receptor negative (ER−) BCC. Prior injections, MCF-7 and T47D cells were cultured 48 h in β-estradiol (E2) 1 nM. All BCC were labeled with 4 µg/ml Fast DiI™ oil red dye and then injected into the perivitelline space of 2 days old zebrafish embryos, which express enhanced green fluorescent protein in endothelial cells. **(A)** MCF-7 cells were injected into zebrafish embryos ± recombinant human IL-8 (rhIL-8) at 1 µg/ml. BCC dissemination was analyzed after 3 days post-injections as described in Section “Materials and Methods,” *n* = 18–20 in each group. **(B)** Western blot analysis of MCF-7 cells treated ± rhIL-8 at 10 ng/ml during 5 days to evaluate the expression of MUC-1, VCAM-1, and ICAM-1. GAPDH load control was reused for illustrative purposes. **(C)** T47D cells were injected into zebrafish embryos ± rhIL-8 at 1 µg/ml. BCC dissemination was analyzed after 3 days post-injections as described in Section “Materials and Methods,” *n* = 17–23 in each group. **(D)** Western blot analysis of T47D cells treated ± rhIL-8 at 10 ng/ml during 3 days to evaluate the expression of MUC-1, VCAM-1, and ICAM-1. GAPDH is shown as load control. **(E)** MDA-MB-231 cells transfected with or without an IL-8 gene silencer RNA were injected into the perivitelline space of zebrafish embryos, and BCC dissemination was analyzed at 3 days post-injections, *n* = 19–22 in each group. **(F)** Western blot analysis of MDA-MB-231 cells transfected with/without an IL-8 silencer RNA (siIL-8) to evaluate the expression of MUC-1, VCAM-1, and ICAM-1. GAPDH load control was reused for illustrative purposes. **(G)** ELISA quantification of extracellular *in vitro* IL-8 in cell culture supernatants of MDA-MB-231 cells transfected with negative control silencer RNA (siRNA Control) or IL-8 silencer RNA (siRNA IL-8) during 2 days showed the successful knockdown of IL-8, *n* = 6 in each group. Representative images of zebrafish embryos are shown. Arrows show disseminated BCC. BV = blood vessels. Results are presented as mean ± SEM, Student’s *t*-test, *p < 0.05, ****p < 0.0001. Western blots shown in this figure were prepared by cropping and pasting from original membranes shown in full in Supplementary Figure 1. Data are representative of at least two independent experiments.

Next, we analyzed whether IL-8 increased BCC dissemination *per se* and if IL-8 affected the expression of cell-adhesion molecules. In the IL-8 low expression cell lines MCF-7 and T47D, addition of human recombinant IL-8 increased BCC dissemination in zebrafish and increased the expression of MUC-1 while ICAM-1 and VCAM-1 remained unaffected (Figures 7A–D). In T47D cells, addition of human recombinant IL-8 slightly decreased ICAM-1 expression; however, this effect might not be relevant for the IL-8-increased dissemination of T47D BCC (Figures 7C,D).

In MDA-MB-231 cells, which express IL-8 at very high levels, we performed an IL-8 gene silencing by RNA of interference. Transfected MDA-MB-231 cells showed a significantly decreased dissemination compared to MDA-MB-231 control cells in zebrafish (Figure 7E). Furthermore, western blot analysis showed that transfected MDA-MB-231 cells lost the expression of MUC-1, ICAM-1, and VCAM-1 integrins (Figure 7F).

## Discussion

Here, we demonstrate a key role of BAd in the early stages of the metastatic cascade of BC *via* IL-8. Up-regulation of IL-8 in the presence of BAd increased the dissemination capacity of BCC by several different mechanisms: (1) by increasing angiogenesis at the primary tumor site, (2) by increasing other cytokines, which in turn enhanced BCC dissemination, (3) by inducing a pro-tumorigenic phenotype of neutrophils, and (4) by modifying the expression of cell-adhesion molecules in BCC and neutrophils. Blocking either IL-8 or VEGF led to reduced angiogenesis at the primary tumor site, but the effect of anti-VEGF on BCC dissemination was limited. In contrast, anti-IL-8 decreased BCC dissemination efficiently. Additionally, we show that the IL-8 levels were 40 times higher than those of VEGF in human BC *in vivo* indeed suggesting that IL-8 is a clinically relevant therapeutic target.

Metastasis is the primary cause of BC mortality. During the first years after BC diagnosis, ER− BC has a higher risk of recurrence. However, after 10 years, the risk of recurrence and death is higher in the ER+ group (24, 25). Since more than two-thirds of all BC are ER+, and more than 25% of these patients will relapse, uncovering the mechanisms underlying the initiation of BCC dissemination is a main concern (26). In addition, the majority of ER+ primary BC maintain the ER expression at the metastatic site (27). One major obstacle for research of the metastatic process of ER+ cells is that all murine BC models that spontaneously metastasize lose ER expression during the dissemination process (i.e., the metastases are ER−). The zebrafish is therefore a crucial model for such research and also excellent for elucidating the role of individual cell types for inducing phenotypical changes of BCC affecting their metastatic capacity (28, 29, 30, 31, 32, 33).

Adipocytes are a major component of breast tissue and contribute to a plethora of growth factors including IL-8 that may affect cancer progression (34, 35). Tumor angiogenesis and inflammation are two of the most important hallmarks for initiation and progression of cancer, and the key proteins for these events are IL-8 and VEGF (36, 37). These two proteins have previously been shown to be interconnected as up-regulation of IL-8 by VEGF and vice versa has been demonstrated in several cancer forms (38, 39). Other proteins may also be involved in BC progression in patients, but our present data support an interconnection of the two proteins *in vivo* as a significant positive correlation between extracellular *in vivo* levels of IL-8 and VEGF were found in BC patients. This was confirmed *in vitro* in co-cultures of ER+ BCC/BAd as anti-IL-8 treatment significantly reduced VEGF secretion. Exposure to E2 further increased the tumor angiogenesis and dissemination of ER+ BCC. This is consistent with data from several groups, which have determined an E2-dependent secretion of VEGF and IL-8 (9, 36, 40, 41, 42).

The interstitial levels of IL-8 are determined by the secretion from all cell types in the tumor microenvironment, such as macrophages, neutrophils, endothelial cells, fibroblasts, adipocytes, and cancer cells (35, 43, 44, 45). The secretion of IL-8 from BCC may be dependent on the microenvironment; BCC that secrete low levels *in vitro* seem to turn on their IL-8 production when grown *in vivo* (46). IL-8 might also be further increased at metastatic sites (47). Thus, cell–cell interactions at the tumor site may affect the expression and secretion of soluble factors. This is in agreement with our present data of mammosphere culture where IL-8 secretion increased but VEGF levels decreased when BCC were co-cultured together with BAd. To the best of our knowledge, this is the first report showing a down-regulation of VEGF by mature adipocytes. However, it has been reported that other cell types such as mesenchymal stem cells may down-regulate VEGF expression in BCC (48). In addition to the concentrations of proteins *in vivo*, the physiological end results may differ dependent on the half-life, affinity to the receptors, and threshold of action of different proteins. Hence, physiologically relevant models and *in vivo* sampling of cytokines are key for studies of complex tissues such as BC.

In the clinic, anti-VEGF therapy has failed as a BC therapeutic (11, 49). We show here that anti-VEGF therapy induced an up-regulation of IL-8 and CCL2 in ER− BCC. This is in line with previous reports of an IL-8 mediated resistance to anti-VEGF therapy in renal cell carcinoma where the tumors were re-sensitized to anti-VEGF therapy by the addition of an anti-IL-8 antibody (50). CCL2 expression has also been correlated to BC progression (51). Our data suggest that a synergistic action of IL-8 and CCL2 by anti-VEGF in ER− BC may be implicated in therapeutic resistance.

CCL5 is another important chemokine that has been correlated with proliferation, migration, invasion, and dissemination of BC by recruitment and a pro-tumoral activation of macrophages (52, 53). CCL5 may be one of the pathways in an IL-8 dependent BCC dissemination as our data indicate that anti-IL-8 treatment significantly reduced the secretion of CCL5. Blocking CCL5 activity led to a significantly reduced ER+ BCC dissemination suggesting that IL-8 may act in a synergistic and co-regulatory fashion with CCL5. These results are in keeping with previous reports showing an interconnection of IL-8 and CCL5 in non-malignant cells (54).

Interleukin-8 is a potent chemokine that may promote cancer metastasis *via* recruitment of neutrophils into the tumor tissue (4). Additionally, neutrophils may mediate the intravasation of BCC *via* increased expression of LFA-1 integrin (12). We show here that conditioned medium from BAd, which contained high levels of IL-8, induced an activation of neutrophils by increased expression of LFA-1 integrin. When neutrophils were injected together with BCC and BAd into the zebrafish, anti-IL-8 and anti-LFA-1 treatments effectively inhibited the BCC dissemination. These results are in agreement with previously reported *ex vivo* data where IL-8 increased LFA-1 integrin in neutrophils, which in turn mediated adhesion to endothelial cells and extravasation of melanoma cells (55).

Besides contributing vast amounts of soluble factors, BAd may establish a unique composition of the ECM. We observed that ER+ BCC acquired the ability to form a tight tumor-like structure in presence of BAd (data not shown). This supports data from pre-adipocytes, which have been shown to secrete a wide variety of proteins such as collagen and laminin during adipogenesis (56). In particular, the presence of adipocyte-derived collagen promotes tumor growth *via* activation of several pathways including IL-8 in the non-metastatic MCF-7 cells (57). This cell–cell contact seems to be needed for the BAd-mediated dissemination; our results revealed that the dissemination of the non-metastatic MCF-7 cells was unaltered when exposed to conditioned medium from BAd but significantly increased by direct cell contact with BAd (data not shown).

Breast adipocyte-induced angiogenesis correlated with the increased dissemination of the otherwise non-metastatic ER+ MCF-7 cells but not with the more aggressive ER+ T47D and ER− MDA-MB-231 cells. It has been reported that T47D cells have higher expression of proteins involved in cancer progression compared to MCF-7 cells including a non-functional form of p53, which increases the invasive and metastatic potential (58, 59). This data indicates that the BAd-induced dissemination might be dependent on the metastatic and invasive capacity of BCC *per se* i.e., BCC with low metastatic capacity may become highly invasive in the presence of BAd and in ER+ BCC, the addition of E2 increases the malignant potential even further. However, pre-adipocytes may affect BCC differently compared with mature adipocytes since our data revealed that pre-adipocytes increased BCC migration regardless of their intrinsic aggressiveness. These data are in line with previous reports showing that pre-adipocytes located in the perivascular space and tumor invasive front have an important role in ER+ as well as in ER− BC metastasis (60).

Overexpression of cell-adhesion molecules in cancer cells promote the interaction with immune- and endothelial cells leading to increased invasion and metastasis (61, 62). High expression of such cell-adhesion proteins has also been reported to correlate with poor prognosis in BC (18, 62, 63). Our data show that IL-8 affected the expression of MUC-1 in ER+ BCC and MUC-1, VCAM-1, and ICAM-1 in ER− BCC. These data corroborate previous reports of an IL-8 dependent up-regulation of ICAM-1 and VCAM-1 in gastric cancer cells (64). Thus, the invasive capacity of BCC *per se* may also be affected by IL-8.

We conclude that BAd modified the tumor microenvironment toward a pro-inflammatory state by increasing the secretion of IL-8, activated neutrophils into a pro-tumoral phenotype, and modified expression of cell-adhesion molecules in BCC and neutrophils. These effects led to increased tumor angiogenesis and BCC dissemination. Our human *in vivo* data revealed very high levels of IL-8, suggesting that IL-8 may be a promising therapeutic target in human BC.

## Ethics Statement

The microdialysis study was performed in accordance with the Declaration of Helsinki, and the Regional Ethical Review Board of Linköping approved the study. All women gave informed written consent. For neutrophils’ isolation from buffy coat, female donors gave informed consent. For animal studies, the Institutional Animal Ethics Committee at Linköping University approved all zebrafish experiments.

## Author Contributions

GR performed the experiments, wrote the article, and analyzed the data, AA performed the experiments and wrote the article, LJ provided the zebrafish embryos, reagents, and equipment for zebrafish experiments and wrote the article, and CD performed the microdialysis investigations, analyzed the data, conceived, designed, and supervised the study, and wrote the Article. All authors have read and approved the article.

## Conflict of Interest Statement

The authors declare that the research was conducted in the absence of any commercial or financial relationships that could be construed as a potential conflict of interest.
