# Supplementary material for: Adipocytes Promote Early Steps of Breast Cancer Cell Dissemination via Interleukin-8
Source: Front Immunol. 2018 Jul 30;9:1767. doi: 10.3389/fimmu.2018.01767 (PMC6077262; doi:10.3389/fimmu.2018.01767)
Supplement: Supplementary file 1 [file data_sheet_1.PDF]

Supplementary Material

Adipocytes promote early steps of breast cancer cell dissemination via interleukin-8

Gabriela Vazquez Rodriguez[1], Annelie Abrahamsson[1], Lasse Dahl Ejby Jensen[2], and Charlotta Dabrosin[1*]

*** Correspondence:** Charlotta Dabrosin charlotta.dabrosin@liu.se

1 Supplementary Figures

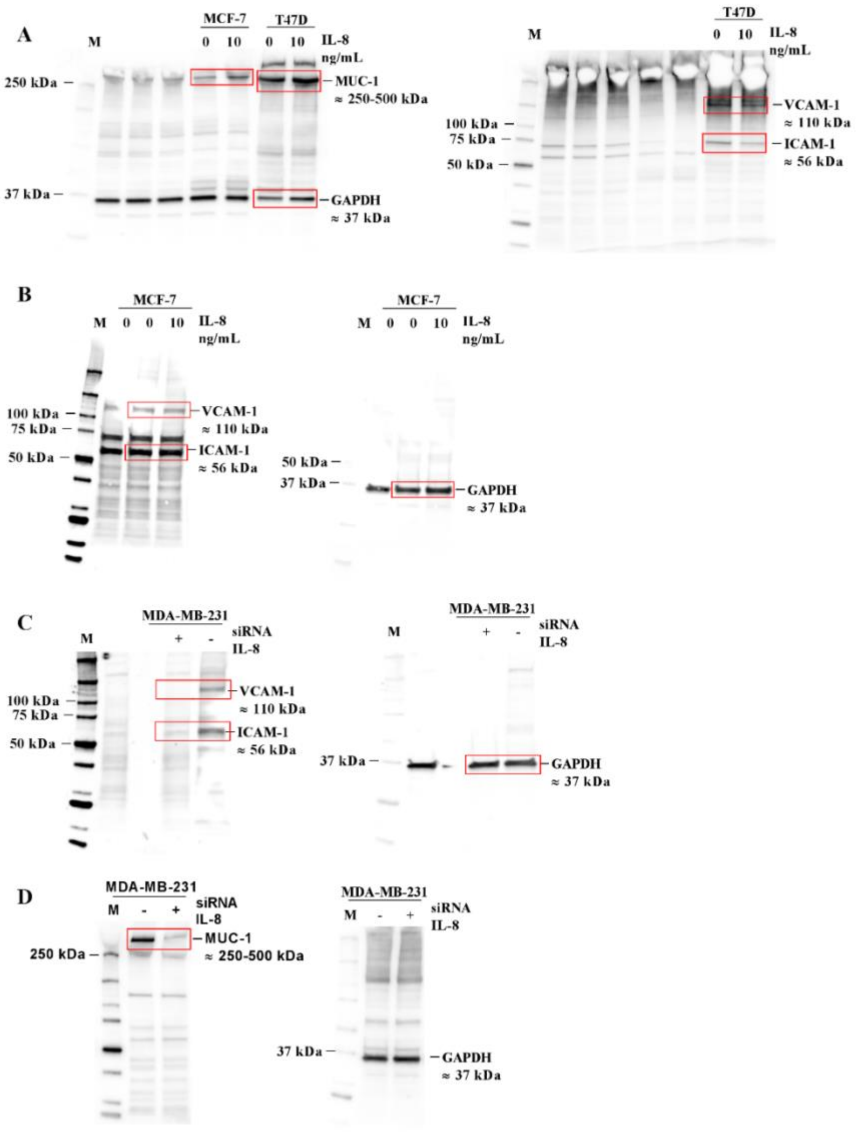

Supplementary Figure 1. Full scan of the entire original membranes of western blot analysis shown in figure 7. (A) Western blot membrane of MCF-7 and T47D cells treated ± rhIL-8 at 10 ng/mL during 5 and 3 days, respectively, to evaluate the expression of MUC-1, ICAM-1 and VCAM-1. GAPDH expression is shown as load control. **(B)** Western blot membrane of MCF-7 cells ± IL-8 at 10 ng/mL during 5 days to evaluate expression of VCAM-1 and ICAM-1. Blot membrane was stripped to show GAPDH expression as load control. **(C)** Western blot membrane of MDA-MB-231 cells transfected ± IL-8 silencing RNA during 2 days to evaluate expression of VCAM-1, ICAM-1 and **(D)** MUC-1. Blot membrane was stripped to show GAPDH expression as load control. Red rectangles highlight the cropped images shown in figure 7. M = molecular weight marker.

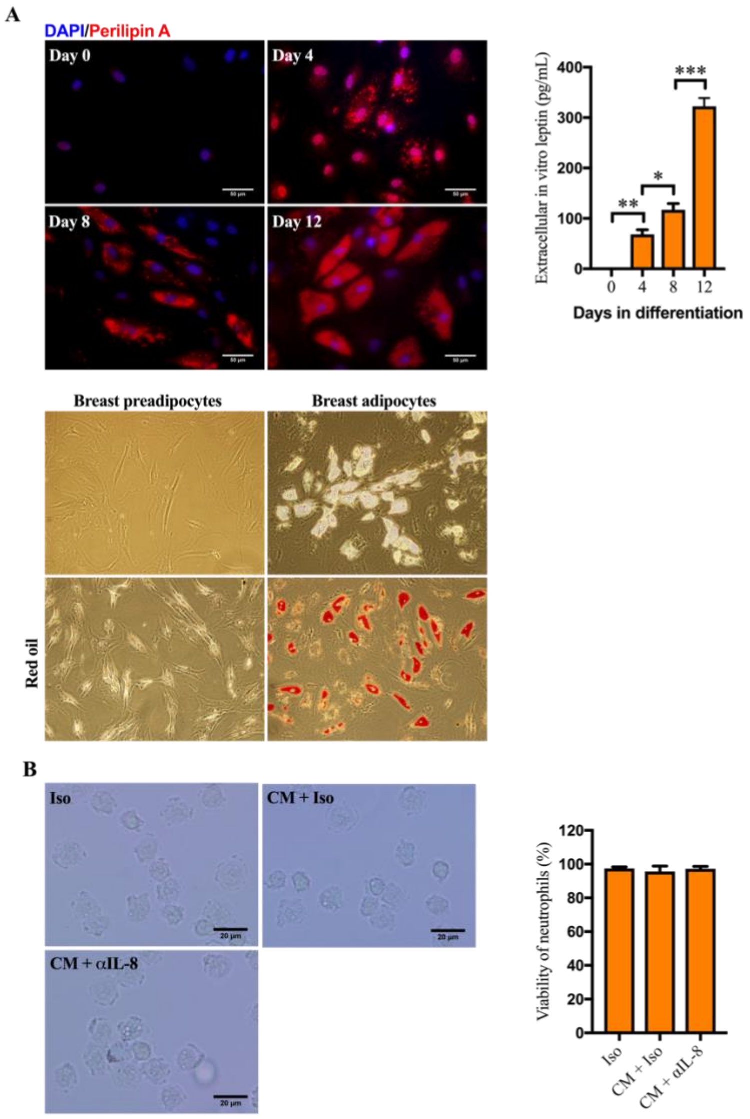

Supplementary Figure 2. Characterization of breast adipocytes differentiation and neutrophils viability assay. For immunocytochemistry and red oil staining, breast preadipocytes were differentiated during 12 and 16 days, respectively, and cover glasses and culture medium samples were taken at 0, 4, 8, 12 and 16 days of differentiation. Neutrophils were cultured at 1 x 10^6 cells/mL in breast adipocytes (BAd)-conditioned or control medium and incubated 45 minutes at 37°C. **(A)** Breast preadipocytes were differentiated during 12 and 16 days. Immunocytochemistry, secreted leptin and red oil staining were analyzed as described in materials and methods, n=3 in each group. Scale bars = 50µm. Bright-field pictures were taken at 20X magnification **(B)** Neutrophils were cultured ± conditioned medium (CM) from BAd ± anti-IL-8 (αIL-8) or isotype control (Iso) at 1µg/mL, and stained with tripan blue as described in materials and methods, n=3 in each group. Results are presented as mean ± SEM, Student *t* test, * $p < 0.05$, ** $p < 0.01$, *** $p < 0.001$.
